# The IRE1α Arm of UPR Regulates Muscle Cells Immune Characters by Restraining p38 MAPK Activation

**DOI:** 10.3389/fphys.2019.01198

**Published:** 2019-09-19

**Authors:** RuiCai Gu, Tao Huang, JiangWei Xiao, ZhaoHong Liao, JunHua Li, HaiQiang Lan, Jun Ouyang, JiJie Hu, Hua Liao

**Affiliations:** ^1^Guangdong Provincial Key Laboratory of Medical Biomechanics, Department of Anatomy, School of Basic Medical Sciences, Southern Medical University, Guangzhou, China; ^2^Department of Orthopaedics and Traumatology, Nanfang Hospital, Southern Medical University, Guangzhou, China

**Keywords:** myotubes, endoplasmic reticulum stress, unfolded protein responses, inositol-requiring enzyme 1α, p38 MAPK

## Abstract

Skeletal muscle repair and systemic inflammation/immune responses are linked to endoplasmic reticulum stress (ER stress) pathways in myopathic muscle, and muscle cells play an active role in muscular immune reactions by exhibiting immunological characteristics under persistent proinflammation stimuli. Whether ER stress affects the intrinsic immunological capacities of myocytes in the inflammatory milieu, as it does to immune cells, and which arms of the unfolded protein response (UPR) mainly participate in these processes remain mostly unknown. We investigated this issue and showed that inflammatory stimuli can induce the activation of the protein kinase R (PKR)-like endoplasmic reticulum kinase (PERK) and inositol-requiring enzyme 1α (IRE1α) arms of the UPR in myocytes both *in vivo* and *in vitro*. UPR stressor administration reversed the increased IFN-γ-induced expression of the MHC-II molecule H2-Ea, the MHC-I molecule H-2K^*b*^, toll-like receptor 3 (TLR3) and some proinflammatory myokines in differentiated primary myotubes *in vitro*. However, further IRE1α inhibition thoroughly corrected the trend in the UPR stressor-triggered suppression of immunobiological molecules. In IFN-γ-treated myotubes, dramatic p38 MAPK activation was observed under IRE1α inhibitory conditions, and the pharmacological inhibition of p38 reversed the immune molecule upregulation induced by IRE1α inhibition. In parallel, our coculturing system verified that the ovalbumin (OVA) antigen presentation ability of inflamed myotubes to OT-I T cells was enhanced by IRE1α inhibition, but was attenuated by further p38 inhibition. Thus, the present findings demonstrated that p38 MAPK contributes greatly to IRE1α arm-dependent immunobiological suppression in myocytes under inflammatory stress conditions.

## Introduction

Potential pathological and physiological disruptors (enhancement of protein synthesis, imbalance in calcium levels, accumulation of misfolded proteins, ischemia, deprivation of glucose or energy, viral infections, hyperhomocysteinemia, or challenge with certain chemicals) will disrupt the homeostasis of the ER and lead to the occurrence of ER stress. The ER engages the UPR to resolve these challenges. ER stress is implemented for the successful initiation of these adaptive responses. If these challenges are not resolved, cells are functionally compromised and activate terminal death processes through inducing ER-related Caspases, JNK, CHOP, or other pathways (Guo et al., 2015).

The ER stress response is involved extensively in the pathological states of various metabolic diseases (e.g., diabetes and obesity) (Kim et al., 2015; Lenin et al., 2015). However, limited information is reported about ER stress function in muscle physiology and pathology. The ER in muscle tissues is specialized and is called the sarcoplasmic reticulum. The sarcoplasmic reticulum is a calcium depot and modulates the release of calcium during muscle contraction, which means that the sarcoplasmic reticulum has a significant role in myofibrillar contraction and is involved in maintaining the homeostasis of muscles (Paran et al., 2015; Nozdrenko et al., 2016). In mouse skeletal muscles, the initiation of the UPR was reported after a high-fat diet consumption and was indicated by the enhanced expression of membrane-bound transcription factor protease site 2, IRE1α, and BiP in the soleus and tibialis anterior muscles (Deldicque et al., 2010). Acute stress in skeletal muscle, such as long distance running, also induces the activation of the ER stress by increasing the expression of spliced XBP-1 and BiP (Kim et al., 2011). Under the condition of cancer cachexia, the PERK pathway is essential for protecting against the loss of muscle mass and strength (Bohnert et al., 2016; Gallot et al., 2019). However, XBP-1 is actived by TLR/MyD88 and leads to skeletal muscle wasting in mice with Lewis lung carcinoma (LLC) (Bohnert et al., 2019). These reports indicate that the ER stress response is one of the primary responses that reacts to the changing environment immediately in muscle tissues. The response may monitor the synthesis of proteins directly and in turn regulate physiological adaptation of skeletal muscles.

In autoimmune myositis, such as sIBM, the expression of the ER chaperones GRP 94, calnexin, GRP78, calreticulin, and ERp72 was enhanced in the pathological muscle area (Vattemi et al., 2004). Increased levels of GRP78 and other ER stress-related genes, including GADD153, protein kinase R (PKR)-like endoplasmic reticulum kinase (PERK) and ATF3, were also observed in muscle biopsies from PM and DM patients (Nagaraju et al., 2005). More specifically, in myositis muscle, increased GRP94 expression was observed in regenerating muscle fibers. Myofibers containing an upregulation of GRP78 and calreticulin also had positive staining for MHC class I in myositis muscle (Nagaraju et al., 2005). Therefore, in myopathic tissue, muscle repair processes and systemic immune responses are both related to the ER stress mechanism.

In physiological conditions, the levels of MHC molecules in muscle cells are not detectable. However, both MHC-I and II have been reported to be expressed in myofibers upon chronic inflammation (such as in polymyositis) (Graca and Kouyoumdjian, 2015). *In vitro*, human myoblasts and myotubes can express MHC classic molecules (HLA-DR), either constitutively or after coculture with IFN-γ. The TLRs (TLR3/TLR7), adhesion molecules (e.g., intercellular adhesion molecule 1), and expression of some B7-related costimulatory/regulatory molecules can also be induced in muscle cells (Wiendl et al., 2005; Ding et al., 2018). This suggests that myocytes are able to present immunological properties and are actively involved in muscle immune/inflammatory responses and could serve as antigen-presenting cells in a proinflammatory environment. In antigen-presenting cells (APCs), ER stress was required for increasing proinflammatory cytokine transcription and influencing the quality of the T cell response induced *in vivo* (Mahadevan et al., 2010; Li et al., 2017). In the myogenic cell line C2C12, the UPR can be induced by the ER stress inducers TM and TG or by palmitic acid *in vitro* (Kwak et al., 2016; Takahashi et al., 2017). A recent report showed that the UPR intensity is related to the intrafiber accumulation of MHC-I molecules, which indicates that immune molecules contribute to ER stress in myofibers and hence may interfere with the immune behaviors of myofibers in myopathic muscle by disrupting ER homeostasis (Freret et al., 2013). Whether ER stress affects the intrinsic immunological capacities of myofibers in the inflammatory milieu, as it does for immune cells, and which branches of the UPR mainly participate in these processes, remains mostly unknown.

In this study, we showed that the IRE1α and PERK arms of the UPR were activated in regenerated myocytes in the CTX-damaged TA muscle. Studies in primary cultured and differentiated myotubes further demonstrated that under proinflammatory stimuli, the UPR, mainly the IRE1α arm, played an important role in regulating the expression of immunobiological molecules in myotubes by attenuating p38 MAPK activity. Our work indicates that, under a persistent proinflammation state, the IRE1α-p38 MAPK signaling axis directly contributes to regulating the immunological behaviors of myotubes.

## Materials and Methods

### Mouse Strains

C57BL/6 (B6) mice were obtained from the Animal Experimentation Centre of the Southern Medical University. All mice were bred and maintained in the pathogen-free condition on a 12-h light/dark cycle and were fed food and water *ad libitum*. TCR-Tg OT-I mice were purchased from the Jackson Laboratory. The TCR of the CD8^+^ OT-I T cells recognizes the chicken ovalbumin peptide OVA257–264 (SIINFEKL) and presents in the context of H-2K^*b*^ MHC class I. All procedures involving the mice were approved by the Southern Medical University Animal Care and Use Committee.

### Animal Experiments

For preparing CTX-induced myoinjury, adult (6–8 week old) wild female B6 mice were injected 50 μL CTX (50 μg/mL; Sigma, United States) into the TA muscles. For inhibiting UPR, the mice were given an injection of 4-PBA (100 mg/kg body weight, i.p.) daily until the end of the experiment. Mice were sacrificed at day 4, 7, 10, or 14 after CTX-induced myoinjury. TA muscle specimens were collected and snap frozen for gene and protein analysis. For histology, muscle specimens were directly snap frozen in liquid nitrogen-cooled isopentane.

### Total RNA Preparation and Transcriptome Microarray

After cervical dislocation, bilateral TA muscles were rapidly dissected out and dipped immediately into RNAlater (RNeasy Protect Mini Kit, Qiagen, Germany) to protect RNA from degradation. The muscles were weighted and incubated overnight at 4°C, then transferred to TRIzol and deep freezed at −80°C prior to further analysis. Muscle samples were then homogenated thoroughly, and incubated the lysate with TRIzol at least 5 min at room temperature (RT). RNA extraction was performed using the phenol/chloroform method and the RNeasy Mini Kit (Qiagen Dusseldorf, Germany) according to the manufacturer’s instructions. Nanodrop (Nanodrop Technologies, United States), RNA Nano kit and RNA 6000 Nano Assay Bioanalyzer (Agilent, United States) were used to analyze the quality/quantity of extracted total RNA, according to the manufacturer’s protocol.

A mouse one color GE 4 × 44K G4846A, V2 microarray kit (G4140-90040, Agilent Technologies, Palo Alto, CA, United States) was used for global transcriptional expression analysis using muscle total RNA samples. RNA labeling, hybridizations, and scanning were performed following the manufacturer’s protocols. Briefly, total RNA (200 ng) was amplified and Cy3-tagged with the Low RNA Input Linear Amplification Kit PLUS, One Color (Agilent) along with Agilent’s One-Color RNA Spike-in Kit according to the manufacturer’s protocols. After the labeling, the cRNA was checked with the NanoDrop Spectrophotometer to evaluate the concentration and quality of the labeling. Each sample (1.65 μg) was hybridized to the custom designed stickleback array at 65°C overnight (17 h) using Agilent’s GE Hybridization Kit. Washes were performed as suggested by the manufacturer using Agilent’s Gene Expression Wash Pack without any stabilization or drying solution. Arrays were scanned with Agilent Technologies Scanner. Spot intensities and other quality control features were extracted with Agilent’s Feature Extraction. Array quality was estimated through the use of Agilent control features as well as spike-in controls (Agilent one-Color Spike-in Kit for RNA experiment). Processed signals from the Feature Extraction Software were used for the analysis. Agilent’s Feature Extraction software automatically normalizes within arrays, subtracts the background, and flags any outlier spots, either due to saturation or non-uniformity. Further information can be learned from the Feature Extraction user guide http://www.chem.agilent.com. Signal ratios of each spot on all slides were normalized by the software and finally, the log ratio was computed as the ratio of the normalized signal of the injured muscles to that of normal muscles. These results were presented as -fold increase or decrease, which are averages of the four independent experiments.

### Cells Culture and Pro-inflammatory Stimuli

According to the previous description, murine MPCs were collected from the hind limb muscle of neonatal B6 mice (Sciorati et al., 2015). MPCs were cultured in Dulbecco’s modified Eagle’s medium Nutrient/F12 (DMEM/F12, Hyclone, United States) containing with 10% fetal bovine serum (FBS, Gibco, United States), 100 U/mL penicillin, and 100 μg/mL streptomycin sulfate in a 5% CO_2_-humidified chamber (Heraeus, Germany) at 37°C. For differentiation studies, MPCs were cultured in differential medium (DM) (DMEM, added with 2% horse serum) for 72 h. For pro-inflammatory stimuli, MPCs were treated with IFN-γ (60 ng/mL, R&D, United States) for 24 h (Nozaki et al., 2010). For inhibiting UPR and the branches, the cells were co-cultured with 4-PBA (10 mM, Selleck, China), 4μ8c (50 μM, Selleck, China), and GSK2606414 (1 mM, Selleck, China), respectively, under pro-inflammatory stimuli. TM (1 μg/mL, Santa Cruz, United States), or TG (0.2 mmol/L, Santa Cruz, United States) were used for 4 h as positive control for testing UPR activation and as UPR activators. Cells were cultured in DM with or without 20 mM SB202190 (Sigma, United States) to blocking p38 activation in IFN-γ medium.

### RNA Extraction and Quantitative Real-Time PCR Analysis

After cervical dislocation, bilateral TA muscles were rapidly dissected out and dipped immediately into RNAlater (RNeasy Protect Mini Kit, Qiagen, Germany) to protect RNA from degradation. The muscles were weighted and incubated overnight at 4°C, then transfered to TRIzol and deep freezed at −80°C prior to further analysis. Total RNA from MPCs was extracted using TRIzol reagent (Invitrogen, United States), following the instructions provided by the manufacturer. 1 μg RNA was then used for reverse transcription (RT) with a commercially available kit (Revert Aid First Strand cDNA Synthesis Kit, Fermentas; PrimeScript^TM^ RT reagent Kit With gDNA Eraser, TaKaRa). Real-time polymerase chain reaction (PCR) was performed in triplicate with an ABI Step One Plus system (Applied Biosystems, United States) and a fluorescence-labeled SYBR Green/ROX qPCR Master Mix kit (Fermentas) adding specific primers. *ATF4, GRP78, GRP94, ATF6, eIF2α, CHOP, XBP-1S, IL-1*β*, IL-6, MCP-1, MIP-1*α, and with glyceraldehyde-3-phosphate dehydrogenase (*GAPDH*) taken as an endogenous control (primer sequences are listed in Table 1), were detected. The relative expression of different gene transcripts was calculated using the ΔΔCt method. The Ct of any gene of interest was normalized to the Ct of GAPDH. Fold changes (arbitrary units) were presented as 2-ΔΔCt.

**TABLE 1:** Gene primer sequences.

**Gene**	**Primer sequence (5′ to 3′)**
*CHOP*	Sense primer: CAGCGACAGAGCCAGAAT Anti-sense primer: AGGGACGGAAAGTGGAAC
*XBP-1S*	Sense primer: GAGTCCGCAGCAGGTG Anti-sense primer: GTGTCAGAGTCCATGGGA
*GRP78*	Sense primer: GTGTGTGAGACCAGAACCGT Anti-sense primer: TCGCTGGGCATCATTGAAGT
*GRP94*	Sense primer: GACCTTCGGGTTCGTCAGAG Anti-sense primer: CTGTCCTTGAGCCTTCTCGG
*ATF6*	Sense primer: TGATGGCTGTCCAGTACACA Anti-sense primer: GCAGATGATCCCTTCGAAAT
*ATF4*	Sense primer: CCTTGTAAGACACCGGAAAT Anti-sense primer: TAGAGATCGTCCTAAAGGC
*eIF2α*	Sense primer: AATCAATGTCGCTAACAAGG Anti-sense primer: TAAAGTTGTAGGTTAGGCGT
*IL-1β*	Sense primer: CACCTTTTGACAGTGATGAG Anti-sense primer: CACAATGAGTGATACTGCCT
*IL-6*	Sense primer: GGTCTTCTGGAGTACCATAG Anti-sense primer: AGCTTATCTGTTAGGAGAGC
*MCP-1*	Sense primer: AAGCTGTAGTTTTTGTCACC Anti-sense primer: AATGTATGTCTGGACCCATT
*MIP-1α*	Sense primer: CTGCCCTTGCTGTTCTTC Anti-sense primer: CAAAGGCTGCTGGTTTCA
*GAPDH*	Sense primer: CTCTGCTCCTCCCTGTTC Anti-sense primer: CAATCTCCACTTTGCCACT

*All primers were designed from mouse sequences using the software Primer Premier 5.*

### Western Blot Analysis

Cell or tissue protein extraction was performed following the manufacturer’s protocol (KeyGEN, China). The following antibodies were used for detection: Mouse monoclonal anti-TLR3 (1:1000, NOVUS, United States), Rabbit polyclonal anti-TLR7 (1:1000, NOVUS, United States), Rabbit polyclonal anti-MHC Class I (H-2K^*b*^, 1:1000, Abcam, United States), Mouse monoclonal anti-MHC-II (H2-Ea, 1:400, Santa Cruz, United States), Rabbit polyclonal anti-IRE1α (phospho Ser724) (1:2000, NOVUS, United States), Rabbit polyclonal anti-IRE1α (1:1500, NOVUS, United States), Rabbit polyclonal anti-p-eIF2α (phospho Ser51) (1:500, Abcam, United States), Mouse monoclonal anti-eIF2α (1:1000, Abcam, United States) Mouse monoclonal anti-ATF6 (3 μg/mL, NOVUS, United States), Rabbit polyclonal anti-Phospho-NF-κB p65 (Ser536) (1:1000, Cell Signaling Technology, United States), Rabbit polyclonal anti-NF-κB p65 (1:1000, Cell Signaling Technology, United States), Rabbit polyclonal anti-Phospho-Erk1/2 (Thr202/Tyr204) (1:1000, Cell Signaling Technology, United States), Rabbit polyclonal anti-Erk1/2 (1:1000, Cell Signaling Technology, United States), Rabbit polyclonalanti-p-p38 (Tyr 182) (1:500, Santa Cruz, United States), Rabbit polyclonalanti-p38 (1:500, Santa Cruz, United States), Phospho-SAPK/JNK (Thr183/Tyr185) Rabbit mAb (1:1000, Cell Signaling Technology, United States), JNK Rabbit mAb (1:1000, Cell Signaling Technology, United States), Mouse monoclonal anti-GAPDH (1:5000, Fudebio, China). Primary antibodies were incubated for 12 h at 4°C in 5% non-fat dried milk in TBS-T. The membrane was then washed in TBS-T for three times and incubated for 1.5 h at RT with horseradish peroxidase conjugated goat anti-rabbit IgG (1:5000, Fudebio, China) or goat anti-mouse IgG (1:5000, Fudebio, China), in 5% non-fat dried milk in TBS-T. Protein concentrations were assessed using a BCA assay kit (KeyGEN, China). Immunoreactive bands were detected by the ECL detection system (Protein Simple, United States), and densitometric values were analyzed with ImageJ v1.42 software (National Institutes of Health, Maryland, United States). The relative expression of each immunoreactive band was calculated by comparison with GAPDH.

### Histological and Immunofluorescence Detection

Snap-frozen whole TA muscle was transversely cryo-sectioned,with the thickness of 8 μm, and either stained with hematoxylin and eosin or prepared for immunostaining. For *in vitro* immunofluorescent labeling, cells were firstly cultured on microscope cover glass (Nest, China), and then fixed in cold acetone for 10 min, permeabilized with 0.1% Triton X-100 for 10 min before washed twice in PBS and prepared for staining. Samples incubated with Rabbit polyclonal anti-IRE1α (phospho Ser724) (1:500, NOVUS, United States); Rabbit polyclonal anti-p-eIF2α (phospho Ser51) (1:200, Abcam, United States); Mouse monoclonal anti-ATF6 (5 μg/mL, NOVUS, United States), Rat anti-mouse lamnin2α (1:200, Abcam, United States), Rabbit polyclonal to CD3 (1:200, Abcam, United States), CD4 Rat (host) Anti-Mouse (1:200, eBioscience, United States), CD8 Rat (host) Anti-Mouse (1:200, eBioscience, United States), CD11b Rat Anti-Mouse (1:200, eBioscience, United States), respectively. Alexa Fluor 488-conjugated goat anti-rabbit IgG (1:500, Beyotime, China), Cy3-conjugated goat anti-rabbit IgG (1:500, Beyotime, China), Cy3-conjugated goat anti-rat IgG (1:500, Beyotime, China), Alexa Fluor 488-conjugated goat anti-mouse IgG (1:500, Beyotime, China), Alexa Fluor 488-conjugated goat anti-rat IgG (1:500, Beyotime, China), were used as secondary antibodies. Nuclei were counterstained with DAPI (Abcam, United States). Slides were scanned with an Olympus BX51 fluorescence microscope (Olympus, Japan).

### Supernatant Preparation for Myokines Secretion Luminex Assay

Culture supernatants (without FBS) were collected and frozen at −80°C immediately after centrifugation and performed analysis within 1 month. Cytokines were analyzed using Luminex xMap technology with Bio-Rad Bio-Plex 200 apparatus (Bio-Rad Laboratories, Inc., H-Wayen, China). The following cytokines, chemokines, and growth factors were assessed in the cohort samples: Eotaxin, G-CSF, GM-CSF, IL-10, IL-12 (p40), IL-12 (p70), IL-17A, IL-1α, IL-1β, IL-2, IL-3, IL-4, IL-5, IL-6, IL-9, MCP-1, MIP-1α, MIP-1β, RANTES, and TNF-α.

### Flow Cytometry Analysis

Spleens and muscle draining lymph nodes (dLNs) from OT-I female mice were crushed and treated with 1x red blood cell lysis buffer (Invitrogen, United States) for 10 min to generate a single cell suspension. Cells were suspended at 10 × 10^6^ cells/ml in complete DMEM/F12 (with 100 U/mL penicillin, 100 μg/mL streptomycin and 10% FBS) and labeled with PE-conjugated monoclonal antibody against CD8 (BD Biosciences, United States), APC-Cy7 conjugated monoclonal antibody CD3 (eBiosciences, United States) for CD8^+^ T cell sorting by FACS. In some experiments OT-I T cells were labelled with CFSE dye (C34554; Invitrogen, United States) by 10 min incubation at 37°C prior to co-cultured with MPC myotubes in the conditional medium for 72 h to assess cell proliferation in the presence of OVA (3 μg/mL, Sigma, United States). In some experiments APC conjugated monoclonal antibody CD69 after co-cultured with MPC myotubes in the conditional medium for 72 h to assess activation of OT-I T cells in the presence of OVA (3 μg/mL, Sigma, United States). Labeled cells were analyzed with a FACSAria II cell sorter (BD Biosciences, New Jersey, United States) with FlowJo software.

### Cell Sorting and Flow Cytometry Analysis

Damaged TA muscles were collected and minced and then gently digested with 0.2% collagenase at 37°C for 45 min twice. Total cells isolated from muscle homogenate 16 were re-suspended in fluorescence activated cell sorting buffer (Phosphate Buffer 17 Solution, 0.5% bovine serum albumin, 2 mM EDTA) to obtain a single cell suspension. CD4 Monoclonal Antibody (GK1.5)-FITC (eBioscience, United States), CD11b Monoclonal Antibody (M1/70)-PE (eBioscience, United States), CD3 Monoclonal Antibody (17A2)-APC (eBioscience, United States), PE Rat Anti-Mouse CD8 (BD, United States). Labeled cells were analyzed with a FACS Aria II cell sorter (BD, Biosciences, United States) with FlowJo.

### Statistical Analysis

All data are expressed as mean ± standard deviation (SD). One-way ANOVA was used for multiple comparisons (Duncan’s multiple range test) using SPSS ver.19.0 software. *P* values < 0.05 were considered as statistically significant.

## Results

### Activation of the UPR Response in Acutely Damaged Muscle and in Regenerated Myofibers

**FIGURE 1:**
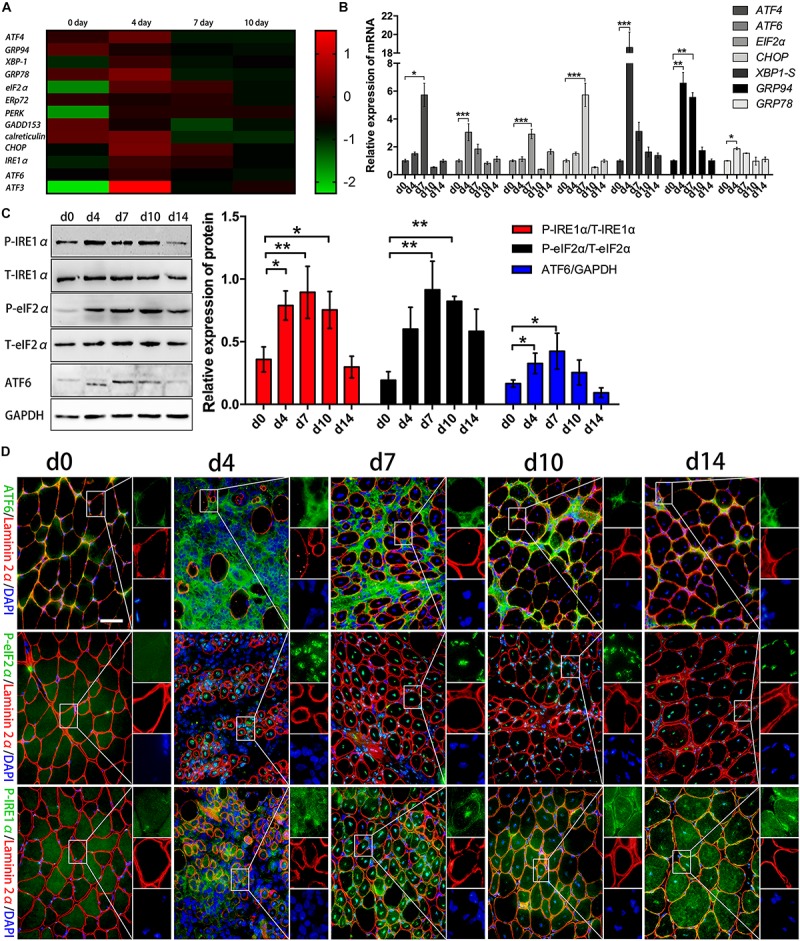
Activation of the UPR response after acute myoinjury. **(A)** RNA-seq analysis demonstrating changes in the mRNA levels of UPR-related genes in damaged TA muscle. **(B)** The mRNA levels of *XBP-1S, ATF4, eIF2*α*, ATF6, GRP78, GPR94*, and *CHOP* in damaged TA muscle were quantified by qRT-PCR. **(C)** Western blot analysis showing protein level changes in P-eIF2α, P-IRE1α, and ATF6 in damaged TA muscle. The relative band intensities from western blot experiments were normalized to the level of T-eIF2α and T-IRE1α or GAPDH. **(D)** Representative immunofluorescence double-staining results of P-eIF2α, P-IRE1α, ATF6 and Laminin2α in damaged TA muscle. All data are presented as the mean ± SD (*n* = 3). One-way ANOVA was used for multiple comparisons (^∗^*p* < 0.05, ^∗∗^*p* < 0.01, and ^∗∗∗^*p* < 0.001). Bar = 50 μm.

Acute myoinjury is related to a self-limited inflammatory response associated with efficient regulatory mechanisms that both favor rapid decrease in postinjury inflammation and promote myorepair (Shi et al., 2018). Using RNA-seq analysis, we monitored a marked gene level increases in some canonical transcription factors of ER stress, and found involvement of *ATF4*, *XBP-1*, *GRP78*, *eIF2*α, *PERK*, *CHOP*, *IRE1α*, *ATF6*, and *ATF3* in acutely damaged TA muscles of B6 mice on day 4 or 7 post injury, compared to healthy muscles (Figure 1A). However, the levels of these transcription factors decreased in the late stage of muscle repair (day 10) (Figure 1A), suggesting that in the degenerating stage and in the early stage of muscle regeneration, the UPR is dramatically activated to cope with damage-induced muscle stress and to tune intramuscular inflammation responses. To ascertain myoinjury-induced ER stress and UPR activation, we further performed qPCR and verified that the expression levels of *XBP-1S, ATF4, eIF2α, ATF6, CHOP, GRP78*, and *GRP94* were elevated in the damaged muscles on day 4 and 7 but were decreased on day 10 and 14 post injury (Figure 1B). In agreement, our western blot analysis further showed that by day 4 and 7 of damage, muscles experienced a rapid increase in phosphorylated eIF2α, IRE1α, and ATF6 (Figure 1C). These data suggested that in response to muscle damage, ER stress may be one of the primary responses that reacts immediately to the inflammatory environment in damaged muscle, which may directly regulate the synthesis of proteins and in turn modulate muscle regeneration. More specifically, immunofluorescence staining (Figure 1D and Supplementary Figure S1) confirmed activation of the UPR in the regenerating muscle fibers of the damaged muscles, as we observed the increased phosphorylation and nuclear translocation of eIF2α and IRE1α in centronuclear myofibers (Figure 1D). Noticeably, we found that ATF6 staining was relatively prominent in the perimysium and fascicle areas (Figure 1D), suggesting that the eIF2α and IRE1α arms of the UPR, but not the ATF6 arm, may directly modulate the protein synthesis of new myofibers in the myoinjury-induced local inflammatory microenvironment.

### The Existence of ER Stress and the UPR in Differentiated Muscle Cells That Received Proinflammation Stimuli *in vitro*

**FIGURE 2:**
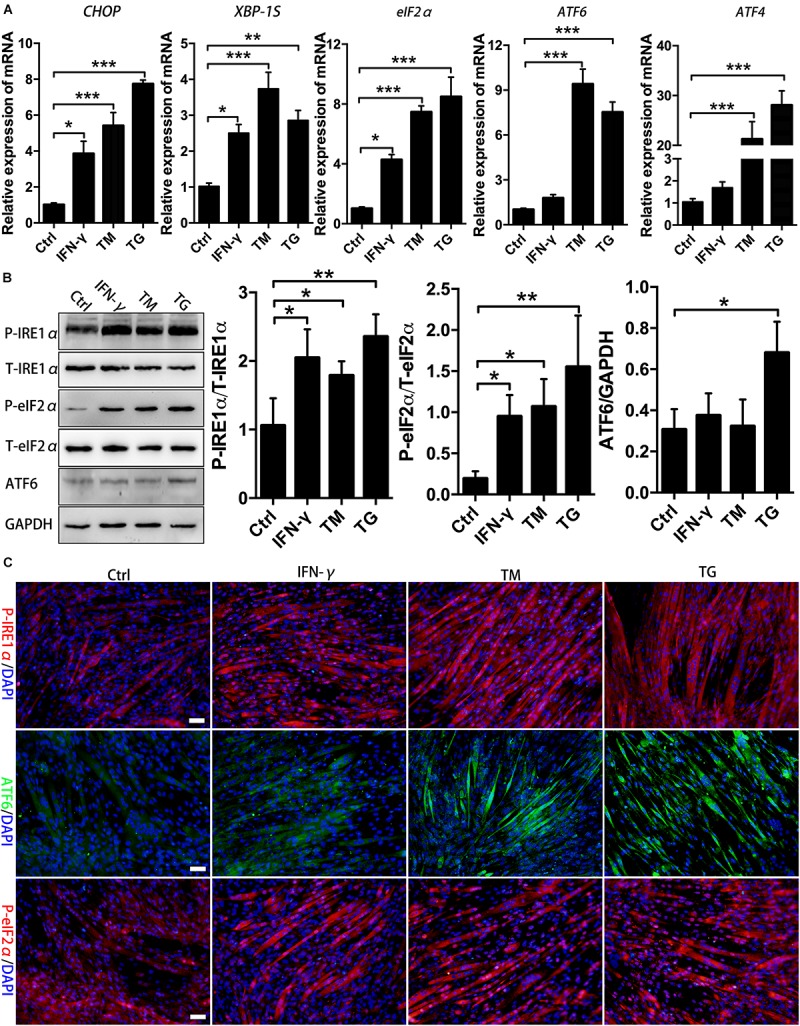
Proinflammatory stimuli trigger ER stress and activate the eIF2α and IRE1α arms of the UPR in MPC-derived myotubes. **(A)** The mRNA expression of UPR markers in TM- or TG-treated myotubes in the presence of IFN-γ, including *CHOP, eIF2α, ATF6, ATF4*, and *XBP-1S*, was quantified by qRT-PCR. **(B)** Western blot analysis showing the protein levels of P-eIF2α, P-IRE1α, and ATF6 in myotubes in the presence of IFN-γ. **(C)** Immunofluorescence staining results of P-eIF2α, P-IRE1α, and ATF6 in IFN-γ-treated myotubes. All data are presented as the mean ± SD (*n* = 3). One-way ANOVA was used for multiple comparisons (^∗^*p* < 0.05, ^∗∗^*p* < 0.01, and ^∗∗∗^*p* < 0.001). Bar = 50 μm.

In muscle cells, the UPR can be induced by metabolic factors (e.g., fatty acid) and exercise-related factors (e.g., PGC-1α) (Woodworth-Hobbs et al., 2017; Kristensen et al., 2018). Recent studies identified UPR markers in regenerating fibers of chronic myositis muscles (Vattemi et al., 2004). We observed the activation of the eIF2α and IRE1α arms of the UPR in centronuclear myofibers in CTX-damaged muscles. In light of our findings, we next sought to identify the UPR arms that are activated in muscle cells under proinflammatory conditions. We cultured and differentiated murine MPCs for 72 h using 2% horse serum, followed by IFN-γ administration for 24 h. Cells were further treated with the ER stress inducers TM and TG or left untreated before the UPR activation analysis. As shown in Figure 2A, TM and TG administration enhanced UPR activities within myotubes, which was indicated by the marked upregulation of *XBP-1S, ATF4, eIF2α, ATF6*, and *CHOP* gene levels compared to those of untreated cells, suggesting that pharmacologic ER stress in muscle fibers employs the UPR and involves the activation of three UPR arms-associated proteins. Interestingly, when we monitored the UPR response in myotubes following proinflammatory stimulation, we found that in the presence of IFN-γ, myotubes markedly upregulated the mRNA levels of *CHOP*, *eIF2*α, and *XBP-1S*, but not for *ATF6* and *ATF4* levels (Figure 2A). By immunoblotting and immunostaining, we also monitored increased protein expression of phosphorylated eIF2α and IRE1α in myotubes in the presence of IFN-γ (Figures 2B,C and Supplementary Figure S2). A recent study has shown that levels of *Ern1* (encoding IRE1) and *Eif2ak3* (encoding PERK), but not *ATF6* (encoding ATF6), are increased in satellite cells upon skeletal muscle injury in adult mice (Xiong et al., 2017). Our data further indicated that to cope with proinflammatory stimuli-triggered ER stress, muscle fibers mainly employ the PERK/eIF2α and IRE1α/XBP-1 arms of the UPR to regulate muscle cell homeostasis and function.

### UPR Inhibition Increased Immunobiological Molecules Production in Myofibers Under Inflammatory Conditions

**FIGURE 3:**
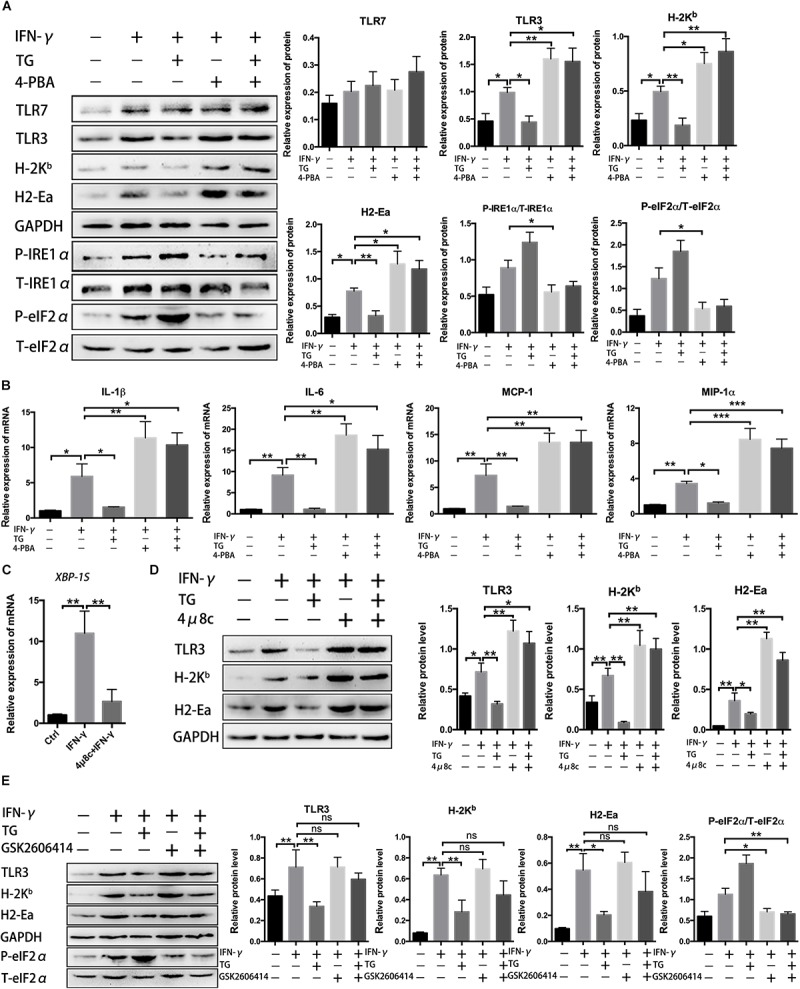
Unfolded protein response inhibition increased immunobiological molecule production in myotubes under inflammatory conditions. **(A)** Western blot analysis showing protein level changes for immune molecules and UPR signal molecules in myotubes exposed to 4-PBA or TG in the presence of IFN-γ. P-eIF2α and P-IRE1α were normalized to T-eIF2α and T-IRE1α, respectively, while H-2K^*b*^, H2-Ea, TLR3, and TLR7 were normalized to the level of GAPDH and analyzed with ImageJ software. **(B)** The mRNA expression of proinflammatory myokines, including *IL-1*β*, IL-6, MCP-1*, and *MIP-1*α, in 4-PBA- or TG-treated myotubes in the presence of IFN-γ, was quantified by qRT-PCR. **(C)** The mRNA expression of *XBP-1S* in IFN-γ treated myotubes incubated with 4μ8c was quantified by qRT-PCR. **(D)** Western blot analysis showing protein level changes for immune molecules in myotubes exposed to 4μ8c or TG in the presence of IFN-γ. H-2K^*b*^, H2-Ea, and TLR3 were normalized to the level of GAPDH and analyzed with ImageJ software. **(E)** Western blot analysis showing protein level changes for immune molecules and P-eIF2α in myotubes exposed to GSK2606414 or TG in the presence of IFN-γ. P-eIF2α was normalized to T-eIF2α. All data are presented as the mean ± SD (*n* = 3). One-way ANOVA was used for multiple comparisons (^∗^*p* < 0.05, ^∗∗^*p* < 0.01, and ^∗∗∗^*p* < 0.001).

Our data thus far support UPR activation in myofibers under the inflammatory conditions both *ex vivo* and *in vivo*. In the inflammatory milieu, muscle cells can secrete critical immunobiological molecules and activate participants in the regulation of local immune recruitment (Wiendl et al., 2005; Ding et al., 2018). Next, we focused our evaluation on the association of UPR activation with the immunological behaviors of myotubes. For that, MPC-derived myotubes that had been differentiated for 72 h were further cultured with the ERS inducer TG and the UPR inhibitor 4-PBA for 24 h, individually or in combination, in the presence of IFN-γ before further analysis. As expected, we first demonstrated that the protein levels of phosphorylated eIF2α and IRE1α in myotubes were markedly enhanced by TG administration and markedly reduced by 4-BPA administration (Figure 3A). Myoblasts and differentiated myotubes have been reported to increase the level of HLA-I (MHC-I) and TLRs or express HLA-II antigen HLA-DR after treatment with IFN-γ *in vitro* (Afzali et al., 2018). Our immunoblotting analysis of primary myotubes supported this point, as we monitored a dramatic elevation in MHC-II (H2-Ea), MHC-I (H-2K^*b*^) and TLR3 protein levels in IFN-γ-treated muscle fibers (Figure 3A and Supplementary Figure S7). Notably, we found that ER stress contributed to suppressing the immunological capacities of myotubes. Under IFN-γ-induced proinflammatory conditions, the addition of TG led to a decrease in protein expression of H-2K^*b*^, H2-Ea, and TLR3 within myotubes. However, this decrease trend was thoroughly corrected by further adding of 4-PBA, resulting in a powerful and significant protein level increase in the abovementioned molecules within myotubes exposed to TG and 4-PBA (Figure 3A). The same trend in expression of the proinflammatory myokine gene levels (*IL-1*β*, IL-6, MCP-1*, and *MIP-1*α) was observed in IFN-γ-treated myotubes upon exposure to TG and 4-PBA (Figure 3B).

Since we already proved that the eIF2α and IRE1α arms of the UPR were activated in myotubes following proinflammatory stimulation, we next evaluated the roles of these two UPR arms on the immune behaviors of myotubes under proinflammatory conditions. To achieve this, we determined the effect of 4μ8c, an IRE1α inhibitor (blocking the endonuclease activity of IRE1α), and GSK2606414, an inhibitor of PERK (reducing eIF2α phosphorylation), on the expression of immunobiological factors within myotubes. IFN-γ-treated myotubes were further incubated for 24 h with TG, 4μ8c or GSK2606414. As expected, we verified that, by blocking IRE1α activity, the 4μ8c treatment decreased *XBP-1S* mRNA levels (Figure 3C and Supplementary Figure S3), while GSK2606414 treatment dramatically reduced myotube expression of phosphorylated eIF2α (Figure 3E). Similar to the effects of 4-PBA (Figure 3A), under inflammatory conditions, myotubes treated with 4μ8c had a significant increase in H-2K^*b*^, H2-Ea, and TLR3 levels compared to those of untreated cells (Figure 3D). Moreover, 4μ8c treatment effectively corrected the TG-induced decrease in immunological molecule levels in myotubes (Figure 3D), indicating that the IRE1α arm of the UPR directly regulates the immune characteristics of myotubes. Notably, we found that eIF2α pathway inhibition by GSK2606414 did not affect muscle fiber immunobiological molecule expression (Figure 3E). Since the eIF2α/PERK arm of the UPR is implicated in regenerative myogenesis (Xiong et al., 2017), we hypothesized that the eIF2α and IRE1α arms of the UPR exert different functions in muscle cells and that IRE1α activation may be related to suppressing the immunological capacities of myotubes.

### IRE1α Affects Immune Characteristics of Muscle Cells by Inhibiting the Activation of p38 MAPK

**FIGURE 4:**
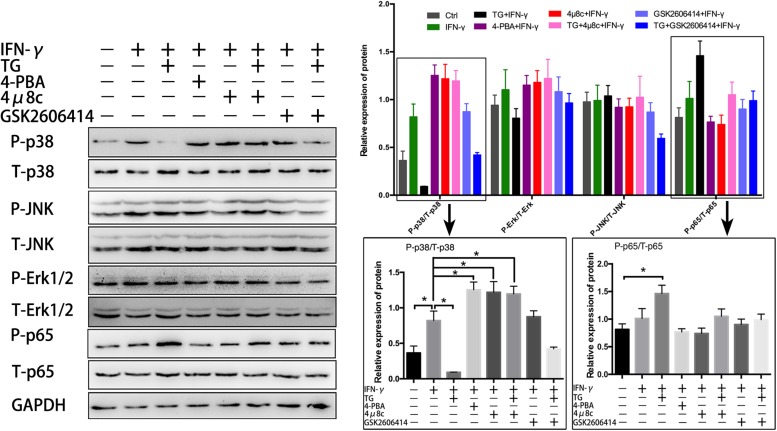
IRE1α inhibition leads to hyperactivation of p38 MAPK in myotubes following proinflammatory stimulation. The NF-κB pathway activation marker P-p65 and the MARK pathway molecules P-p38, P-Erk1/2, and P-JNK were analyzed by immunoblot analysis in IFN-γ treated myotubes, after adding of 4-PBA, TG, 4μ8c, or GSK2606414. The relative band intensities from western blot experiments were normalized to the level of T-p65, T-p38, T-Erk1/2, T-JNK, or GAPDH. All data are presented as the mean ± SD (*n* = 3). One-way ANOVA was used for multiple comparisons (^∗^*p* < 0.05).

It has been shown that activation of IRE1α specifically recruits IκB kinase (IKK) to the ER and then activates NF-κB, leading to proinflammatory cytokine production in immune cells (Yu et al., 2015). In addition, ER stress is reported to crosstalk with MAPK and tune inflammatory signaling pathways and immune cell survival and may contribute to dysregulated inflammatory responses (Bettigole and Glimcher, 2015). To explore the underlying mechanisms by which the UPR attenuates IFN-γ-mediated immunobiological capacities in myotubes, we examined the canonical NF-κB pathway activation marker phospho-p65 (P-p65) and the MARK pathway molecules phospho-p38 (P-p38), phospho-Erk1/2 (P-Erk1/2) and phospho-JNK (P-JNK) by immunoblot analysis. We found that in IFN-γ-treated myotubes, the expression level of P-p38 but not of P-Erk1/2 and P-JNK, was considerably enhanced compared to that of untreated cells (Figure 4). The P-p38 level was further elevated by adding 4-PBA but was reduced by adding TG (Figure 4); importantly, we did not see IFN-γ-induced P-p65 expression alteration in myotubes, despite increased P-p65 expression in TG-administered myotubes (Figure 4). These data suggested that, under proinflammatory conditions, the UPR response was activated in myotubes, which may further recruit MAPK pathways, mainly p38 signaling, to regulate a series of muscle cell functions. Intriguingly, when we monitored the IRE1α arm in IFN-γ-treated myotubes, we found that 4μ8c treatment further increased the protein level of P-p38 but not of P-JNK, P-p65, and P-Erk1/2. Indeed, 4μ8c treatment reversed the TG-induced P-p38 decrease in inflamed myotubes (Figure 4), suggesting that IRE1α inhibition leads to hyperactivation of p38 MAPK in myotubes following proinflammatory stimulation. This hypothesis was further verified by exploring the effect of the eIF2α pathway inhibitor GSK2606414, as we did not detect significant expression changes for the above-examined molecules in myotubes administered IFN-γ and GSK2606414 compared to myotubes administered IFN-γ alone (Figure 4).

**FIGURE 5:**
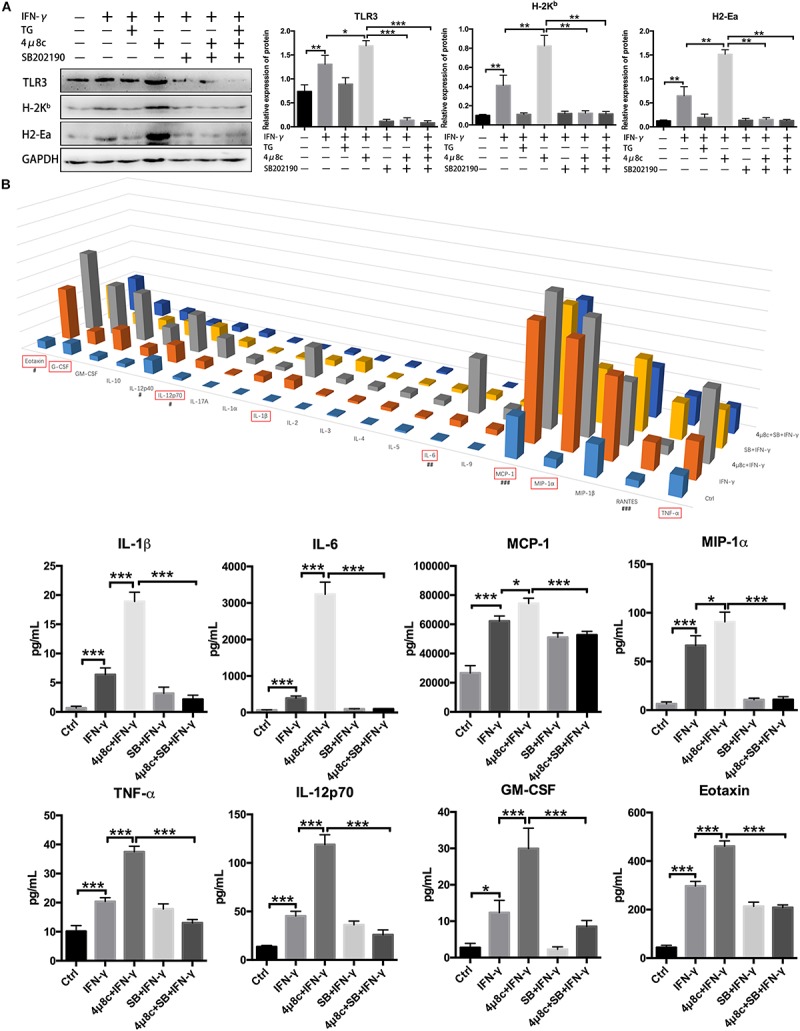
The IRE1α arm directly affects the immune characteristics of muscle fibers by interfering with p38 MAPK activity. **(A)** Western blot analysis showing H-2K^*b*^, H2-Ea, and TLR3 protein levels in muscle cells exposed to IFN-γ, 4μ8c, TG, and SB202190, individually or in combination. The relative band intensities from western blot experiments were normalized to the level of GAPDH and analyzed with ImageJ software. **(B)** Luminex assay showing protein level changes for proinflammatory myokines in muscle cells exposed to IFN-γ, 4μ8c, TG and SB202190, individually or in combination (*^#^*at 0.1 × level; *^##^*at 0.01 × level; *^###^*at 0.001 × level). All data are presented as the mean ± SD (*n* = 3). One-way ANOVA was used for multiple comparisons (^∗^*p* < 0.05, ^∗∗^*p* < 0.01, and ^∗∗∗^*p* < 0.001) (SB = SB202190).

To further clarify that the effects of IRE1α on myotube immune characteristics are relevant to p38 MAPK activity, we cotreated myotubes with 4μ8c and SB202190, an inhibitor of p38 MAPK (inhibition of p38 phosphorylation), under proinflammatory conditions. Western blot analysis showed that the addition of SB202190 could thoroughly reverse the 4μ8c-mediated increase in protein levels of H-2K^*b*^, H2-Ea and TLR3 in myotubes exposed to IFN-γ (Figure 5A). Using a Luminex assay, we further discovered that the protein levels of some proinflammatory myokines, including IL-1β, IL-6, MCP-1, MIP-1α, TNF-α, IL-12p70, GM-CSF and Eotaxin, which are highly expressed in muscle fibers in response to IFN-γ administration or IFN-γ and 4μ8c coadministration, were significantly decreased upon coincubation with SB202190 (Figure 5B). Taken together, these results suggest that the IRE1α arm of the UPR is activated in muscle cells under persistent proinflammatory stimuli and directly affects the immune characteristics of muscle fibers through further interfering with p38 MAPK activation.

### The IRE1α Arm of the UPR Is Necessary for Maintaining Immunologic Role of Muscle Cell

**FIGURE 6:**
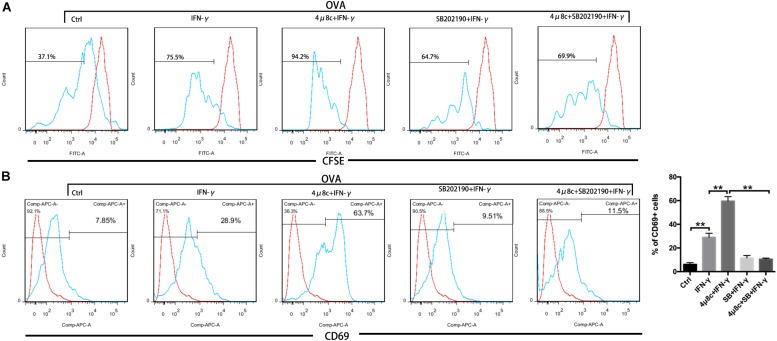
The IRE1α arm of the UPR regulates myotube immune capacity by attenuating p38 MAPK activity. CFSE-labeled OVA-specific OT-I T cells were cocultured with MPC-derived myotubes, pretreated with IFN-γ, 4μ8c, and SB202190, individually or in combination. FACS analysis of the CFSE fluorescence loss **(A)** and CD69 expression **(B)** on OT-I cells. Data are presented as the mean ± SD (*n* = 3). One-way ANOVA was used for multiple comparisons (^∗∗^*p* < 0.01) (SB = SB202190).

Muscle fibers are intrinsically capable of antigen processing and presentation (APC function) under persistent treatment with IFN-γ (Ding et al., 2018). Our results so far have demonstrated that the IRE1α arm of the UPR regulates myotube immune behaviors. To ascertain whether the IRE1α axis directly interferes with the APC capacity of myotubes, we next turned to coculture of myotubes and CD8^+^ T cells. For this, purified CFSE-labeled OVA-specific CD8^+^ T cells from TCR-Tg OT-I mice were cocultured with MPC-derived myotubes that were pretreated with IFN-γ, 4μ8c, and SB202190, individually or in combination. OVA protein was then added to coculturing system. OT-I cell activation and proliferation were detected by FACS analysis of CD69 expression and the fluorescence loss of CFSE, individually. As expected, in the presence of OVA, OT-I cell priming can be induced by coculturing with IFN-γ-treated myotubes but not by coculturing with untreated myotubes (Figure 6), suggesting that the antigen-presenting capacity of muscle cells was triggered by IFN-γ stimulation, resulting in presentation of OVA antigen to OT-I cells. Interestingly, we found that OT-I cell priming was markedly enhanced by pretreatment with 4μ8c but was suppressed by pretreatment with SB202190. Moreover, we detected OT-I cell priming induced by 4μ8c, which was reversed by SB202190 treatment (Figure 6). In a parallel experiment, we did not detect any significant difference in OT-I cell priming between the GSK2606414-treated and 4-PBA-treated coculturing systems (data not shown). Combined, these data indicate that the IRE1α arm of the UPR regulates myotube-mediated immune efficacy by attenuating p38 MAPK activity.

### UPR Inhibition Increases the Immunity Infiltration in Injured Muscle Tissues

**FIGURE 7:**
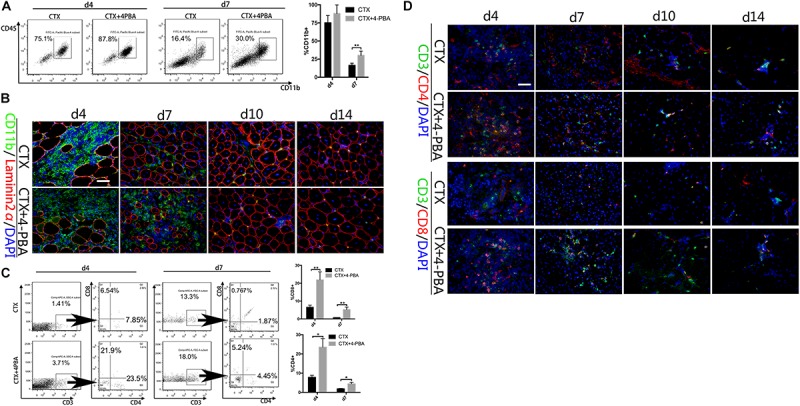
4-PBA treatment increased immune cells in injured muscle. **(A)** FACS analysis of the proportion of CD11b^+^ cells in CD45-gated cells isolated from TA muscle on day 4 and 7 post injury in 4-PBA + CTX-treated mice and CTX-treated mice. **(B)** Representative immunofluorescence double-staining results of CD11b and Laminin2α in damaged TA muscle. **(C)** FACS analysis of the proportion of CD8^+^ cells and CD4^+^ cells in CD3^+^-gated cells isolated from TA muscle on day 4 and 7 post-injury in 4-PBA + CTX-treated mice and CTX-treated mice. **(D)** Representative immunofluorescence double-staining results of P-eIF2α, P-IRE1α, ATF6, and Laminin2α in damaged TA muscle. All data are presented as the mean ± SD (*n* = 3). One-way ANOVA was used for multiple comparisons (^∗^*p* < 0.05 and ^∗∗^*p* < 0.01). Bar = 50 μm.

To further access the effect of the UPR on the interactions between myocytes and immune cells *in vivo*, we analyzed monocytes/macrophages (F4/80^+^ or CD11b^+^) and T cells (CD8^+^ or CD4^+^) in CTX-injured TA tissues. Cytokines produced by myocytes and other cells in injured muscle tissues can induce monocyte/macrophage chemotactic movement into the injury area. As shown in Figure 7, 4-PBA treatment increased the percentage of CD11b^+^ cells during the acute inflammatory reaction stage and muscle tissue regeneration stage (4 and 7 days) (Figures 7A,B). However, F4/80^+^ cells showed no difference between the CTX-injured group and the CTX + 4-PBA group (data not shown). Antigens expressed by muscle cells induce the migration of T cells into the endomysium or perimysium during myositis. Acute muscle injury also resulted in transient infiltration of T cells. In this study, application of 4-PBA increased the percentage of CD4^+^ and CD8^+^ T cells in muscle tissues compared to that of CTX-treated mice (Figures 7C,D). On the basis of these results, we conclude that inhibiting the UPR increases the immune infiltration in damaged muscle tissues, which indicates the enhanced immune responses of muscle tissues.

## Discussion

Cellular stress responses triggered exogenously or endogenously cause accumulation of misfolded proteins in the ER lumen and the consequent activation of the UPR, which enables cells to either resolve the stress or initiate apoptosis. The UPR has three main effectors: IRE1α, PERK, and ATF6. In response to the accumulation of unfolded/misfolded proteins, IRE1α, PERK, and ATF6 are dissociated and activated to enhance the ER protein folding capacity by upregulating chaperone protein levels and reducing global protein synthesis (Bettigole and Glimcher, 2015). IRE1α is a transmembrane protein residing in the ER membrane, with endonuclease and kinase activities. Upon activation, IRE1α causes splicing of XBP-1 mRNA. As one part of the UPR, the IRE1α-XBP-1 axis monitors and maintains the fidelity of the cellular proteome (Medel et al., 2018; Bohnert et al., 2019). Recent studies have shown that the IRE1α arm of the UPR plays an important role in the development, differentiation, and precise function of immune cells. For example, cellular stress, such as low oxygen or glucose levels and ionic pressure, enhanced the generation of Th17 cells by increasing cytoplasmic calcium levels and XBP-1 activity. In contrast, inhibition of cellular stress and conditional deletion of XBP-1 in lymphocytes suppressed Th17 cell-dependent autoimmunity in an EAE mouse model (Deslauriers et al., 2011); the IRE1α pathway was activated by acute infection and was required for T cell differentiation into effector T cells (Kemp et al., 2013); In macrophages, IRE1α is a positive regulator of TLR responses, and TLR2 and TLR4 activated the IRE1α–XBP-1 axis but not the PERK or ATF6α pathways. Moreover, the IRE1α pathway was required for macrophages producing proinflammatory cytokines, such as IL-6, TNF and INF-β (Shi et al., 2009; Qiu et al., 2013; Reverendo et al., 2019). In dendritic cells (DCs), the IRE1α pathway has been demonstrated to have developmental and survival roles, as XBP-1-deficient chimeric mice had decreased numbers of conventional and plasmacytoid DCs (Reverendo et al., 2019).

Numerous studies have reported that the UPR is activated in both acute and chronic inflammation. TLR ligation specifically activates the IRE1α/XBP-1-signaling axis while suppressing the PERK signaling branch directly, thereby enhancing proinflammatory cytokine production at the expense of canonical UPR target genes such as PDI and ERdj4 (Martinon et al., 2010). Skeletal muscle tissue is a hotbed for the immune response. Muscle immune reactions develop spontaneously during the process of infectious muscle diseases and autoimmunity or are induced by muscle gene therapy (Prud’homme et al., 2001). The initiation of ER stress in the pathogenesis of muscle inflammation diseases, such as PM and DM, has been reported (Vattemi et al., 2004). Here, our work demonstrated the dramatic activation of the UPR involving eIF2α, IRE1α, and ATF6 in acutely damaged muscle tissue during the muscle inflammation process, implying immune roles for muscle UPR via direct crosstalk between ERS-induced signaling pathways and the immune response.

Numerous studies have reported that muscle cells in the immune process can act as active participators rather than passive onlookers that express various immunological molecules to maintain and amplify local inflammatory immune reactions (De Rossi et al., 2000; Nagaraju, 2001; Wiendl et al., 2005). Our recent work (Ding et al., 2018), with data described by others (Nagaraju, 2001; Wiendl et al., 2005), supports the intrinsic immune properties of muscle cells in inflammatory conditions. Despite the description of ER stress in the response to muscle metabolic consequences, acute stressors, and cancer cachexia (Bohnert et al., 2016, 2019; Woodworth-Hobbs et al., 2017; Kristensen et al., 2018), including autoimmune and genetic muscle disorders (Afroze and Kumar, 2017), the role of the individual arms of the UPR in regulating muscle fiber immune function has not been fully explained. Our results with CTX-treated mice provide evidence that the eIF2α and IRE1α arms of the UPR are critical for muscle fiber function during skeletal muscle regeneration. Recent research emphasized that the PERK branch of the UPR plays a significant role in regulating muscle satellite cell survival and expansion during skeletal muscle regeneration. Genetic deletion of XBP-1 in satellite cells had little effect on skeletal muscle regeneration. The author thus suggests that the IRE1α/XBP-1 branch of the UPR is not necessary for satellite cell-mediated regenerative myogenesis (Xiong et al., 2017). Since we detected the activation of the eIF2α and IRE1α arms in regenerated centronuclear myofibers, we reasoned that the IRE1α arm of the UPR is presumably associated with other functions of muscle cells, such as immune capacities.

On the basis of previous reports, ER stress-associated responses are mainly involved in proinflammation actions (Reverendo et al., 2019). However, we found that UPR or IRE1α inhibition causes a further increase in the levels of immunobiological molecules in IFN-γ-treated myotubes; in comtrast, TM and TG treatment reduced the production of these molecules, suggesting that persistent ER stress suppresses the IFN-γ-mediated immune efficiency of myotubes. This view is in line with the recent observations (Ho et al., 2012) that ER stress negatively modulated innate immunity caused by LPS and IFNs. In APCs, such as mucosal and lung DCs, deletion of XBP-1 resulted in disturbance of ER architecture, downregulated expression of CD11c and inability to cross-present dead cell-associated antigens but did not produce an effect on cell survival. In contrast, the IRE1-XBP-1 axis is important for the suppression of antitumor immunity. XBP-1 promotes the triglyceride biosynthetic program in DCs, which leads to abnormal lipid accumulation and impaired antigen presentation. Conversely, DC-specific deletion of XBP-1 restored immunostimulatory activities and increased antitumor responses (Osorio et al., 2014). These data highlight that in different microenvironments, the IRE1α arm of the UPR may have distinct roles in regulating immune cell function. Our results demonstrate that the administration of the pharmacological stressors TM and TG, the UPR pathway inhibitor 4-BPA, and the specific IRE1α inhibitor 4μ8c do not affect muscle fiber survival and differentiation. Interestingly, we found that treatment with 4μ8c elevated muscle fiber immune molecule levels and promoted myotube OVA antigen presentation to OT-I cells. This finding is further evidence that the IRE1α branch of the UPR is critical for myotubes exerting immunobiological function and that IRE1α contributes to downregulating immune capacities in muscle cells. From our data, it is possible that, after acute myoinjury, the IRE1α arm of the UPR is activated to inhibit the production of immunobiological molecules in regenerated myofibers, which in turn protects muscle from the muscle fiber-mediated immune reactions and prompts the muscle repair.

Endoplasmic reticulum stress has been reported to be associated with classical inflammation pathways, including NF-κB and MAPKs (Erk, JNK and p38) (Bettigole and Glimcher, 2015). Notably, ER stress does not always initiate the activation of NF-κB signaling. Low-dose ER stress preconditioning actually decreases TNF-α-induced NF-κB activation in endothelial cells, thereby attenuating the inflammatory upregulation of cell adhesion molecules (Li et al., 2011). A previous study also demonstrated IRE1α-dependent activation of MAPK pathways. Phosphorylated IRE1α can directly activate JNK by sequentially recruiting TRAF2 and ASK1. Due to the persistent activation of IRE1α, unremitting JNK activation drives inflammation and proinflammatory chemokine production in tissue (Bettigole and Glimcher, 2015). At later stages of myogenesis, p38 MAPK is activated to initiate a muscle gene expression program (Xiong et al., 2017). We did not find a significant change in the P-p65 and MARK pathway molecules P-Erk1/2 and P-JNK in myotubes pretreated with 4μ8c, under proinflammatory conditions. Conversely, we monitored IRE1α inhibition leading to the notable activation of p38 in myotubes in the presence of IFN-γ. Furthermore, inhibition of p38 reversed the 4μ8c-induced upregulation of immune molecules in myotubes and attenuated the proliferation of OT-I cells cocultured with myotubes pretreated with 4μ8c. These data imply that p38 MAPK contributes greatly to IRE1α arm-dependent immunobiological regulation in myotubes under inflammatory stress conditions.

The immune character exhibited by skeletal muscle cells allow skeletal muscle cells to be actively involved in inflammatory responses (Afzali et al., 2018). The *in vivo* results showed UPR inhibition and showed that the infiltration of immune cells was obviously increased in the injured muscle area. These results are consistent with the *in vitro* outcomes and further confirmed the inhibitory role of the UPR in myocyte immune behavior.

In summary, our study shows initial evidence that the IRE1α branch of the UPR is essential for suppressing the immunological capacities of muscle fibers under proinflammatory conditions. Our findings provide the basis for evaluating IRE1α activity in muscle diseases; as in mouse models of acute muscle injury, increased IRE1α activity may prove beneficial for the inhibition of muscular immune recruitment triggered by regenerated muscle fibers.

### Study Limitation

The primary skeletal muscle cells were treated only with inhibitors or agonists because the cells were very sensitive and could not be transformed with shRNA or siRNA, which reduced the power of this research. It also remains to be determined whether the specific deficiency of UPR pathways in skeletal muscle has an effect on myocyte immune behaviors *in vivo*. The use of chemical reagents *in vivo* may affect the microenvironment of skeletal muscle tissues by affecting a variety of cells that are resident or translocating into muscle tissues. The use of transgenic mice and a variety of muscle injury methods are needed for further study.

## Data Availability

All datasets for this study are included in the manuscript and/or the Supplementary Files.

## Ethics Statement

Animal experiments were approved by the local institutional ethic committee for animal experimentation.

## Author Contributions

HL and JH conceived the project. RG, TH, JX, ZL, and HQL collected the data. RG, TH, JX, JO, JL, and HQL analyzed the data. HL, RG, and TH wrote the manuscript.

## Conflict of Interest Statement

The authors declare that the research was conducted in the absence of any commercial or financial relationships that could be construed as a potential conflict of interest.

## Supplementary Material

FIGURE S1 The fluorescent intensity of ATF6, P-eIF2α, and P-IRE1α in injured skeletal muscle tissues at 0 day, 4 day, 7 day, 10 day, and 14 days (^∗^*p* < 0.05, ^∗∗^*p* < 0.01 and ^∗∗∗^*p* < 0.001).

FIGURE S2 The fluorescent intensity of ATF6, P-eIF2α, and P-IRE1α in primary myotubes (^∗^*p* < 0.05 and ^∗∗^*p* < 0.01).

FIGURE S3 The mRNA level of XBP-1S in primary myotubes under the stimulation of different concentration of 4μ8c for 24 h; the viability of primary myotubes under the stimulation of different concentration of 4μ8c for 24 h (^∗^*p* < 0.05, ^∗∗^*p* < 0.01 and ^∗∗∗^*p* < 0.001).

FIGURE S4 The protein expression of RIPK in myotubes under the stimulation of IFN-γ, GSK2606414, or IFN-γ+GSK2606414 for 24 h.

FIGURE S5 The GAPDH bands and ponceau s staining in gradient dilution myotubes lysates.

FIGURE S6 H&E and Dystrophin/DAPI IF staining in muscle fibers of TA muscle. Bar = 100 μm.

FIGURE S7 The survival of IFN-γ-treated-myofibers incubated with TG, 4μ8c or GSK2606414, respectively, was demonstrated by MyHC staining and under phase contrast microscopy. Bar = 50 μm.

## Abbreviations

APCs: antigen presenting cells
CHOP: C/EBP-homologous protein
CTX: cardiotoxin
DM: dermatomyositis
EAE: experimental autoimmune encephalomyelitis
ER: endoplasmic reticulum
GRP: glucose regulatory protein
IRE1 α: inositol-requiring enzyme 1 α
JNK: c-JunN-terminal kinase
MHC: major histocompatibility complex
MPCs: myogenic precursor cells
PM: polymyositis
sIBM: sporadic inclusion body myositis
TA: tibialis anterior
TG: Thapsigargin
TLRs: toll-like receptors
TM: Tunicamycin
UPR: unfolded protein response
XBP-1: x-box binding protein 1.
