# Supplementary figures and images for: The IRE1α Arm of UPR Regulates Muscle Cells Immune Characters by Restraining p38 MAPK Activation
Source: Front Physiol. 2019 Sep 19;10:1198. doi: 10.3389/fphys.2019.01198 (PMC6761248; doi:10.3389/fphys.2019.01198)

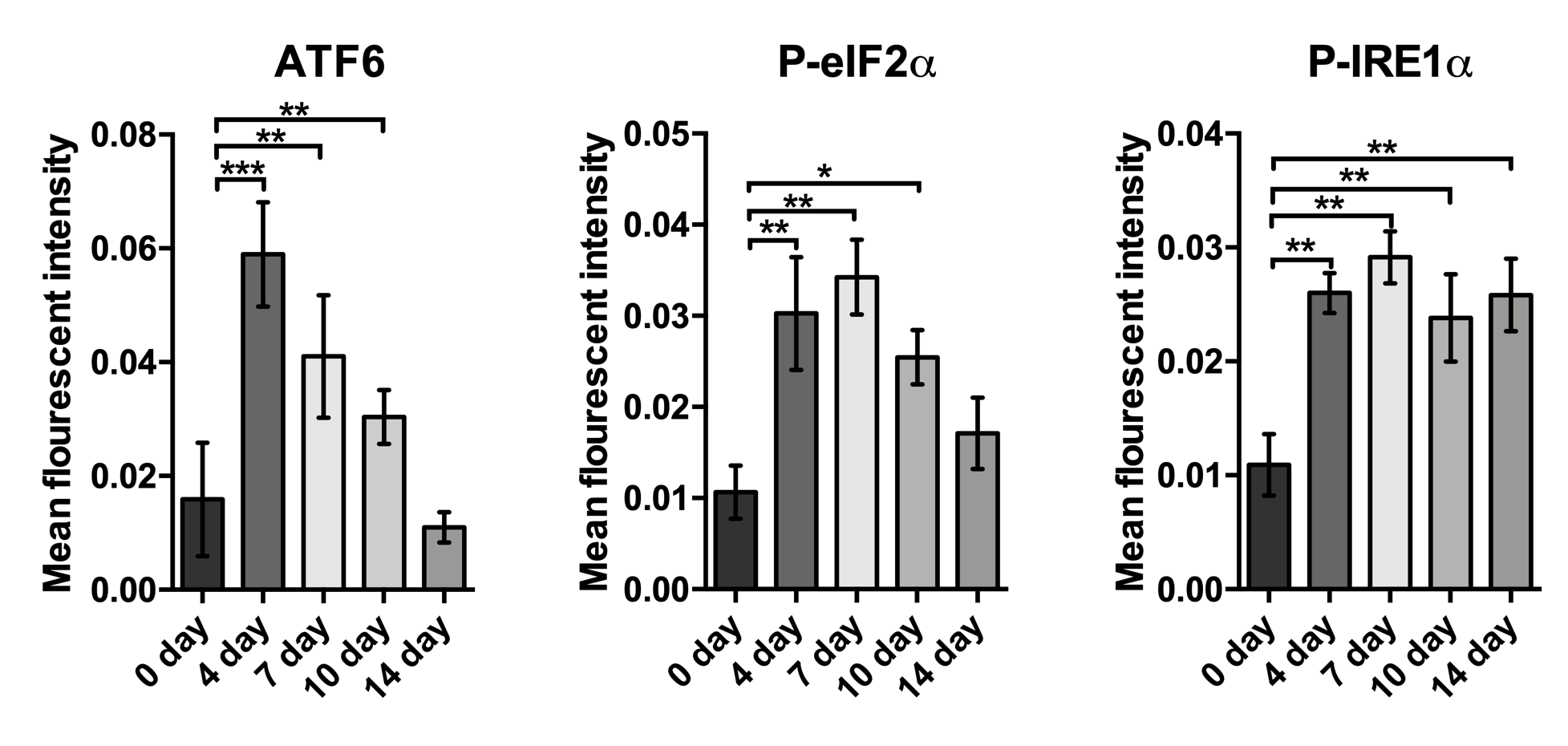

Supplement: FIGURE S1 — The fluorescent intensity of ATF6, P-eIF2α, and P-IRE1α in injured skeletal muscle tissues at 0 day, 4 day, 7 day, 10 day, and 14 days (∗p < 0.05, ∗∗p < 0.01 and ∗∗∗p < 0.001). [file Image_1.TIFF]

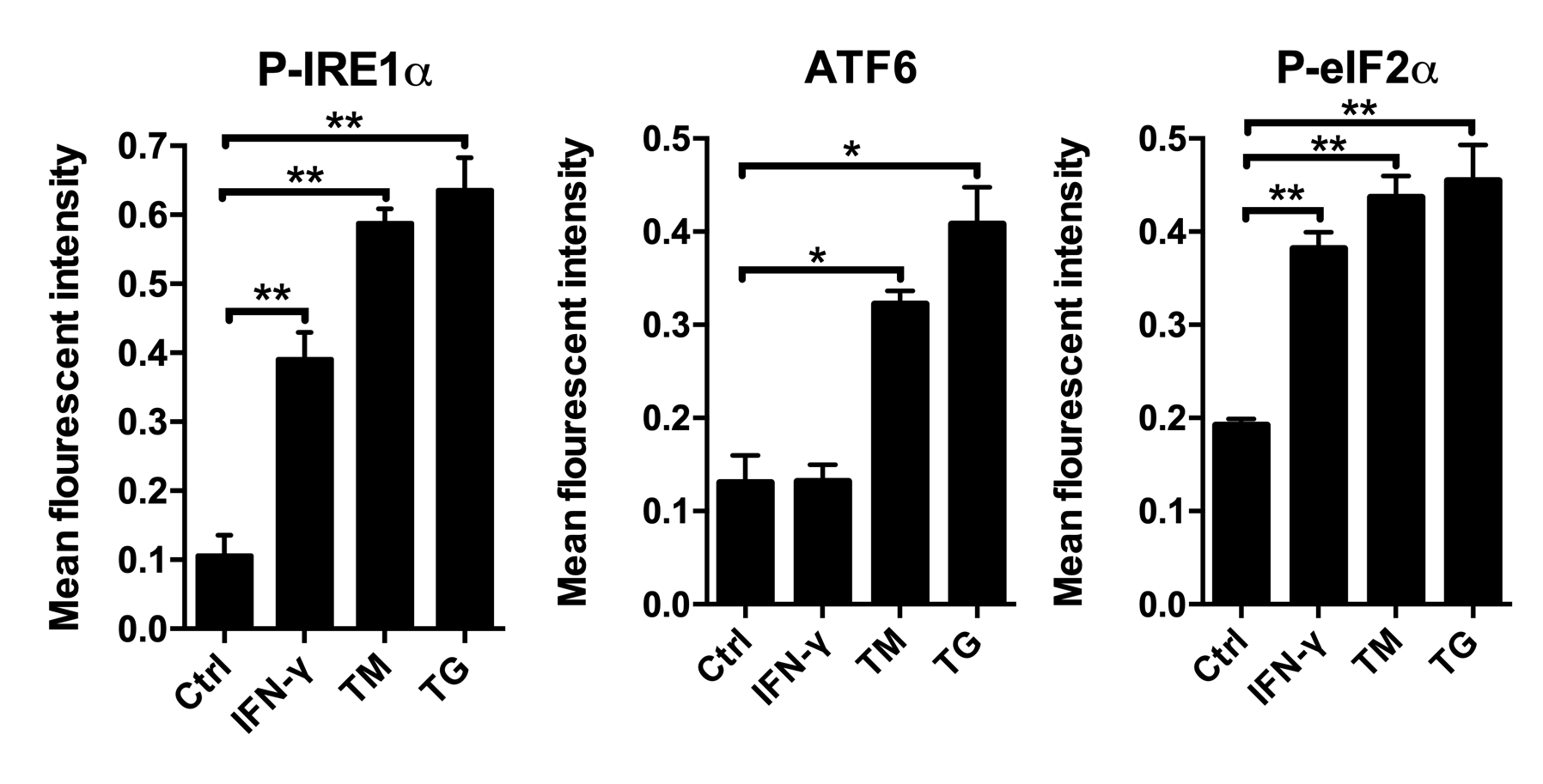

Supplement: FIGURE S2 — The fluorescent intensity of ATF6, P-eIF2α, and P-IRE1α in primary myotubes (∗p < 0.05 and ∗∗p < 0.01). [file Image_2.TIFF]

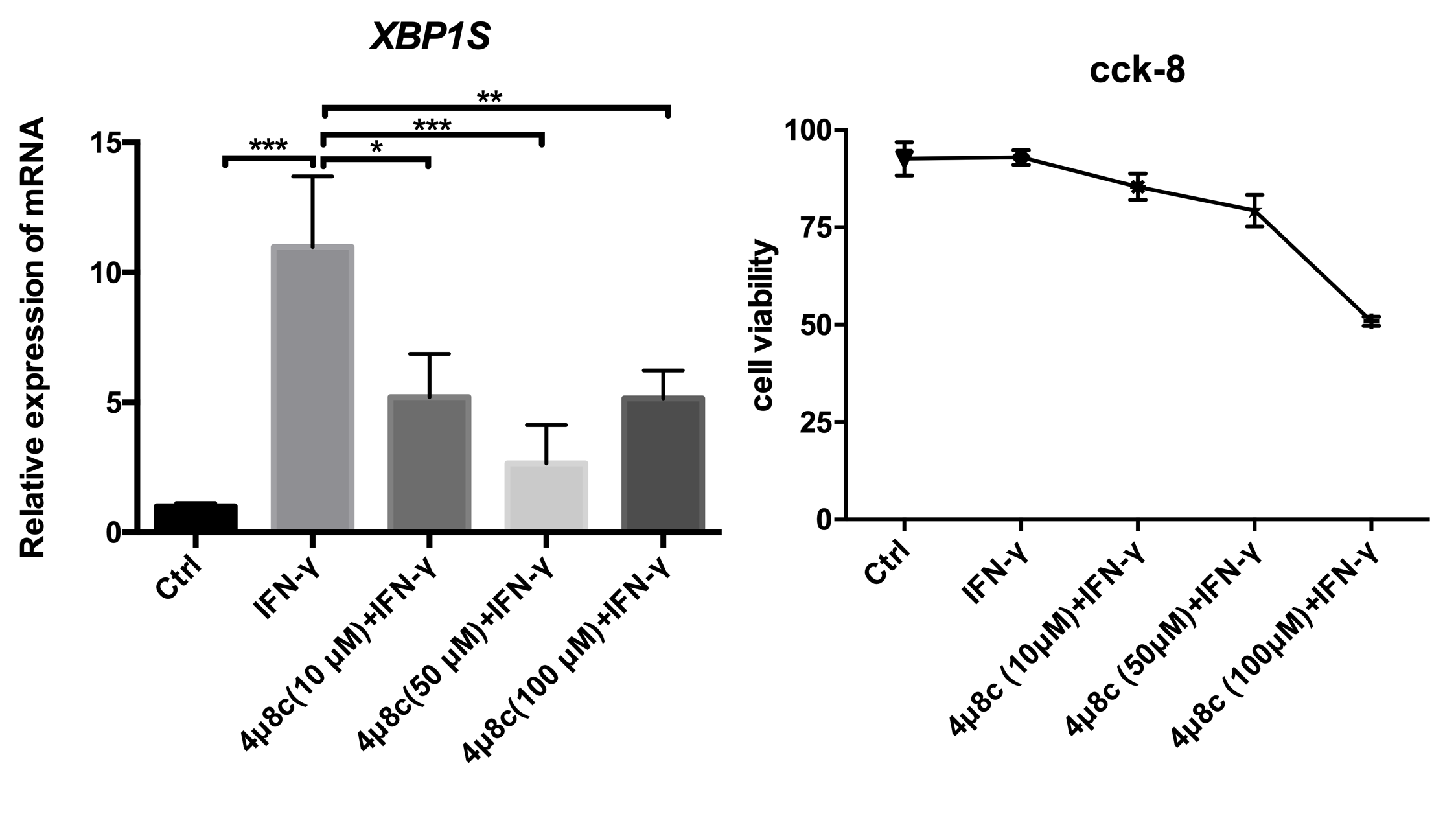

Supplement: FIGURE S3 — The mRNA level of XBP-1S in primary myotubes under the stimulation of different concentration of 4μ8c for 24 h; the viability of primary myotubes under the stimulation of different concentration of 4μ8c for 24 h (∗p < 0.05, ∗∗p < 0.01 and ∗∗∗p < 0.001). [file Image_3.TIF]

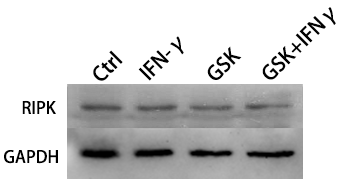

Supplement: FIGURE S4 — The protein expression of RIPK in myotubes under the stimulation of IFN-γ, GSK2606414, or IFN-γ+GSK2606414 for 24 h. [file Image_4.TIF]

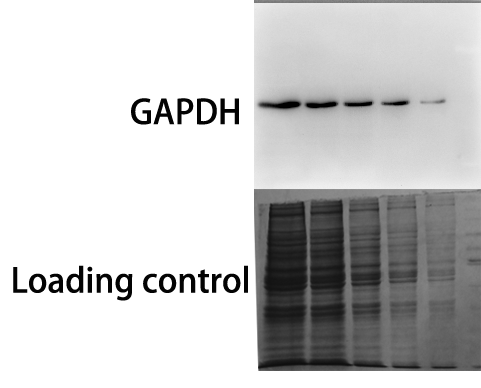

Supplement: FIGURE S5 — The GAPDH bands and ponceau s staining in gradient dilution myotubes lysates. [file Image_5.TIF]

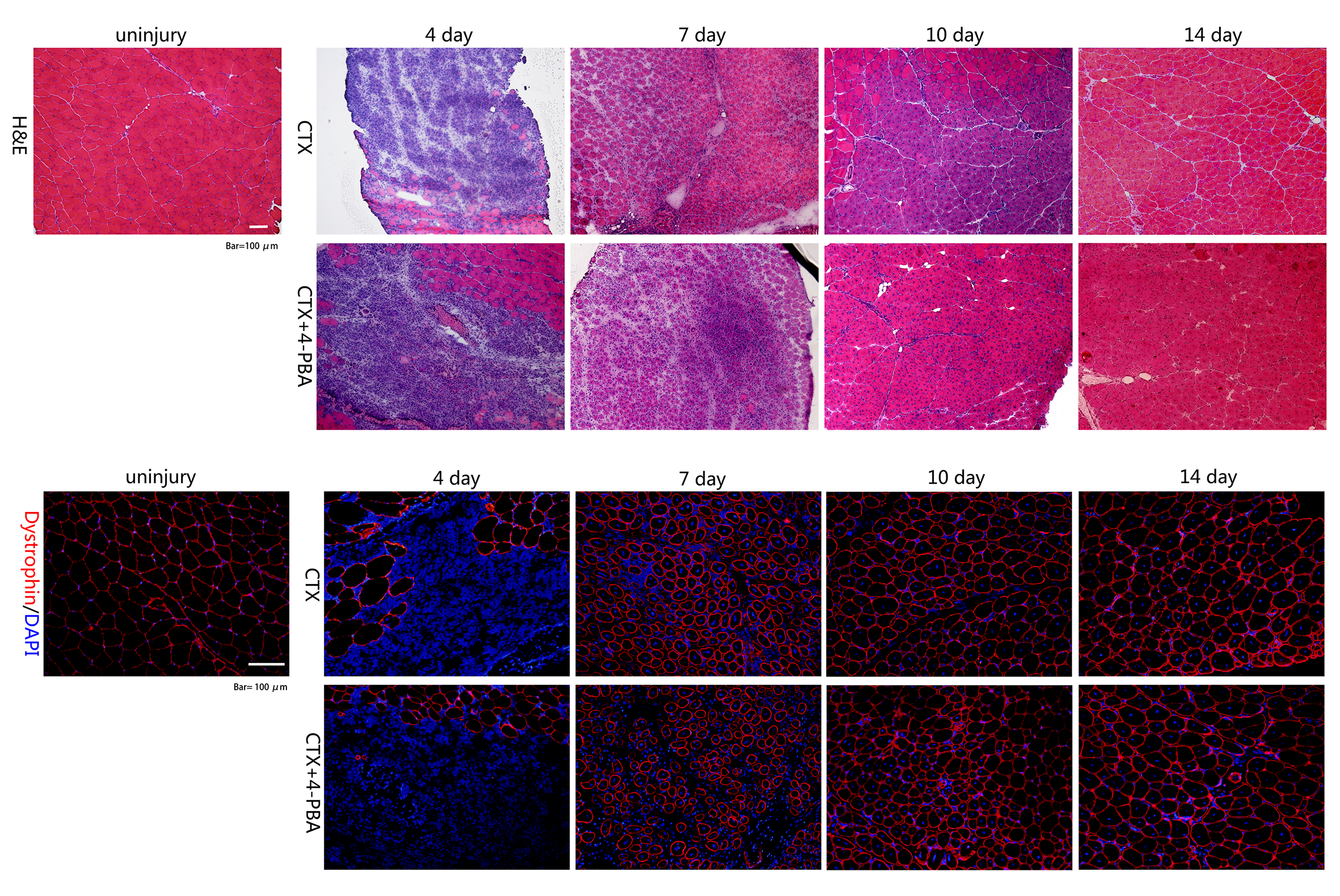

Supplement: FIGURE S6 — H&E and Dystrophin/DAPI IF staining in muscle fibers of TA muscle. Bar = 100 μm. [file Image_6.TIF]

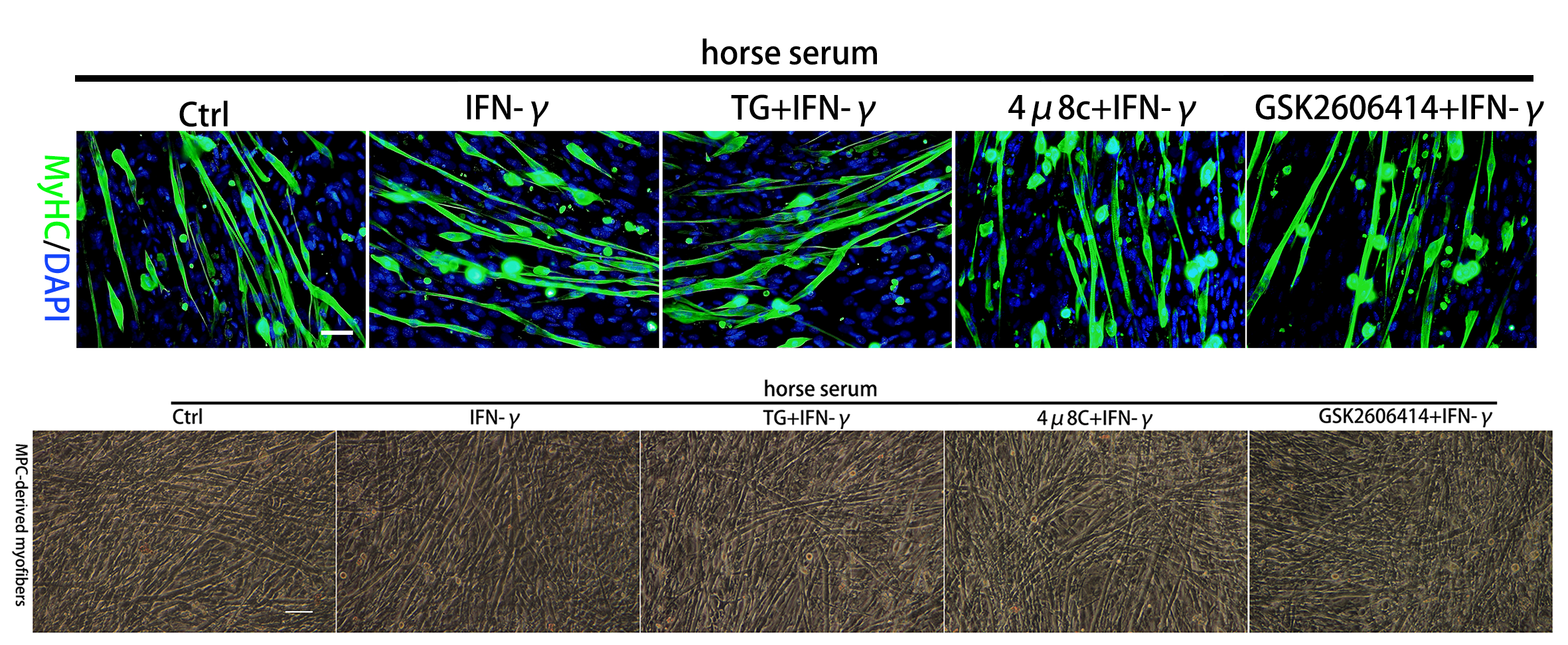

Supplement: FIGURE S7 — The survival of IFN-γ-treated-myofibers incubated with TG, 4μ8c or GSK2606414, respectively, was demonstrated by MyHC staining and under phase contrast microscopy. Bar = 50 μm. [file Image_7.TIF]
